# TSC2/Rheb signaling mediates ERK‐dependent regulation of mTORC1 activity in C2C12 myoblasts

**DOI:** 10.1002/2211-5463.12195

**Published:** 2017-01-30

**Authors:** Mitsunori Miyazaki, Tohru Takemasa

**Affiliations:** ^1^ Department of Physical TherapySchool of Rehabilitation SciencesHealth Sciences University of HokkaidoJapan; ^2^ Graduate School of Comprehensive Human SciencesUniversity of TsukubaIbarakiJapan

**Keywords:** C2C12, extracellular signal‐regulated kinase, mechanistic target of rapamycin complex 1, rapamycin, Ras homolog enriched in brain, tuberous sclerosis 2

## Abstract

The enhanced rate of protein synthesis in skeletal muscle cells results in a net increase in total protein content that leads to skeletal muscle growth/hypertrophy. The mitogen‐activated protein kinase kinase (MEK)/extracellular signal‐regulated kinase (ERK)‐dependent regulation of the activity of mechanistic target of rapamycin (mTOR) and subsequent protein synthesis has been suggested as a regulatory mechanism; however, the exact molecular processes underlying such a regulation are poorly defined. The purpose of this study was to investigate regulatory mechanisms involved in the MEK/ERK‐dependent pathway leading to mTORC1 activation in skeletal muscle cells. Treatment with phorbol‐12‐myristate‐13‐acetate (PMA), a potent agonist of protein kinase C (PKC) and its downstream effector in the MEK/ERK‐dependent pathway, resulted in the activation of mTORC1 signaling and phosphorylation of the upstream regulator tuberous sclerosis 2 (TSC2) in C2C12 myoblasts. PMA‐induced activation of mTORC1 signaling was partially prevented by treatment with U0126 (a selective inhibitor of MEK1/2) or BIX‐02189 (a selective inhibitor of MEK5) and completely blocked with BIM‐I (a selective inhibitor of upstream PKC). TSC2 phosphorylation at Ser664 (an ERK‐dependent phosphorylation site) was prevented with U0126, and BIM‐I treatment blocked PMA‐induced phosphorylation of TSC2 at multiple residues (Ser664, Ser939, and Thr1462). Overexpression of Ras homolog enriched in brain (Rheb), a downstream target of TSC2, and an mTORC1 activator, was sufficient to activate mTORC1 signaling. We also identified that PMA‐induced activation of mTORC1 signaling was significantly inhibited in the absence of Rheb with siRNA knockdown. These observations demonstrate that the PKC/MEK/ERK‐dependent activation of mTORC1 is mediated through TSC2 phosphorylation and its downstream target Rheb in C2C12 myoblasts.

**Abbreviations**
4E‐BP1: eukaryotic initiation factor 4E‐binding protein 1
CT: cycle threshold
DMEM: Dulbecco's modified Eagle's medium
ERK: extracellular signal‐regulated kinase
MEK: mitogen‐activated protein kinase kinase
mTORC1: mechanistic target of rapamycin complex 1
Rheb: Ras homolog enriched in brain
rpS6: 40S ribosomal protein S6
S6K1: p70 ribosomal S6 kinase 1
TSC1: tuberous sclerosis 1
TSC2: tuberous sclerosis 2

The net balance between protein synthesis and degradation is a critical determinant of the regulation of skeletal muscle mass and long‐term human health. The enhanced rates of protein synthesis, which were overcoming protein degradation, result in a net increase in cellular protein accumulation that leads to skeletal muscle growth and hypertrophy 1. It has been well documented that mechanistic target of rapamycin (mTOR) plays an essential role in the regulation of protein synthesis and subsequent cell hypertrophy in skeletal muscle 2, 3, 4, 5. mTOR is a member of the phosphatidylinositol 3‐kinase‐related kinase family and forms two different multiprotein complexes, mTOR complex 1 (mTORC1) and mTORC2 6. mTORC1 is a rapamycin‐sensitive protein complex which contains the catalytic mTOR subunit and associated protein partners, including regulatory‐associated protein of mTOR (Raptor) and G protein β‐subunit‐like 7. In skeletal muscle cells, mTORC1 functions as a central integrator of a wide range of signal inputs including growth factors, nutrients, cellular energy states, and mechanical strain, which then promotes protein synthesis and cell growth/hypertrophy 1. Once activated, mTORC1 enhances protein translation by phosphorylating the two identified downstream effectors, eukaryotic initiation factor 4E‐binding protein 1 (4E‐BP1) and the p70 ribosomal S6 kinase 1 (S6K1). Phosphorylation of 4E‐BP1, a translational repressor, releases it from an inhibitory complex with the translation initiation factor eIF4E, thereby promoting cap‐dependent translation. S6K1 phosphorylates the 40S ribosomal protein S6 (rpS6), leading to active translation of mRNAs 8.

Currently, the most well‐defined signaling mechanism regulating mTORC1 activity in skeletal muscle is the phosphoinositide 3‐kinase (PI3K) and its downstream effector, Akt (also referred as protein kinase B)‐dependent pathway 9. PI3K/Akt‐dependent regulation of mTORC1 activity involves complex, multistep machinery. Briefly, Akt phosphorylates the heterodimeric protein complex, tuberous sclerosis 1 (TSC1) and TSC2, which then leads to the inhibition of its function as a GTPase‐activating protein (GAP) toward Ras homolog enriched in brain (Rheb), a direct mTORC1 activator 10, 11, 12. Akt‐mediated inhibition of TSC2‐GAP activity allows the small G‐protein Rheb to accumulate in its active GTP‐bound form, thereby stimulating mTORC1 activity 10, 11, 13. A well‐known anabolic agent in skeletal muscle, insulin‐like growth factor‐1, has been reported as an effective agonist for activating PI3K/Akt‐dependent pathway 2, 3, 12. Therefore, the PI3K/Akt/mTORC1 cascade was considered as a major regulatory axis for enhancing protein translation and skeletal muscle growth/hypertrophy.

In contrast, it has been also reported that mTORC1 integrates other signal inputs such as nutrients, cellular stresses, and mechanical strain, and these stimuli are mediated independently of PI3K/Akt pathway 1. We have previously reported that mechanical overload‐induced activation of mTORC1 signaling in skeletal muscle is mediated independently of the PI3K/Akt pathway and further provided evidence that the mitogen‐activated protein kinase kinase 1/2 (MEK1/2) and its downstream effector extracellular signal‐regulated kinase 1/2 (ERK1/2) pathway may contribute to mTORC1 activation *in vivo* 14. The contribution of ERK1/2‐dependent signaling to skeletal muscle anabolism has been proposed 15, 16, 17, 18; however, the exact molecular mechanisms underlying this regulation remain poorly understood. The purpose of this study was to determine the potential contribution of MEK/ERK‐dependent pathway leading to mTORC1 activation in skeletal muscle cells.

## Materials and methods

### Materials

C2C12 mouse myoblasts were purchased from ATCC (Manassas, VA, USA). High‐glucose Dulbecco's modified Eagle's medium (DMEM) and FBS were from GIBCO (Grand Island, NY, USA). FuGENE 6 Transfection Reagent was from Roche (Indianapolis, IN, USA). Lipofectamine 2000, TRIzol reagent, SuperScript III First‐Strand Synthesis SuperMix, and TURBO DNA‐free were from Invitrogen (Carlsbad, CA, USA). Protein A Plus Agarose was from Thermo Fisher Scientific (Rockford, IL USA). PMA and rapamycin were from Calbiochem (San Diego, CA, USA). U0126 was from LC Laboratories (Woburn, MA, USA). BIX‐02189 was from Adooq Bioscience (Irvine, CA, USA). Bisindolylmaleimide I (BIM‐I) was from Cayman Chemical (Ann Arbor, MI, USA). Protease inhibitor cocktail for mammalian tissues was from Sigma‐Aldrich (St. Louis, MO, USA). Protein assay dye reagent concentrated was from Bio‐Rad Laboratories (Hercules, CA, USA). Odyssey Blocking Buffer was from LI‐COR Biosciences (Lincoln, NE, USA).

### Antibodies

Phospho‐S6K1 (Thr389), phospho‐S6K1 (Thr421/Ser424), Akt, phospho‐Akt (Thr308), phospho‐Akt (Ser473), TSC1/Hamartin, phospho‐TSC2 (Ser939), phospho‐TSC2 (Ser1387), phospho‐TSC2 (Thr1462), phos‐ERK1/2 (Thr202/Tyr204), ERK1/2, phos‐ERK5 (Thr218/Tyr220), ERK5, phos‐rpS6 (Ser235/236), phos‐rpS6 (Ser240/244), rpS6, phos‐RSK (Thr359/Ser363), phos‐RSK (Ser380), and RSK1/2/3 were from Cell Signaling Technology (Danvers, MA, USA). TSC2/Tuberin (C‐20) and S6K1 (C‐18) were from Santa Cruz Biotechnology (Santa Cruz, CA, USA). GAPDH, cadherin, myc, and phospho‐TSC2 (Ser664) were from Abcam (Cambridge, MA, USA). IRDye 800CW Goat anti‐(mouse IgG) and IRDye 680LT Goat anti‐(Rabbit IgG) secondary antibodies were from LI‐COR Biosciences.

### Plasmid DNA

pRK5‐myc‐Rheb was kindly provided from Dr. Esser's Laboratory, University of Florida, USA.

### Cell culture and transfection

All cell culture experiments were performed in a humidified environment at 37 °C in a 5% CO_2_ atmosphere. The murine skeletal muscle cell line C2C12 myoblasts were grown in DMEM supplemented with 10% FBS, and penicillin–streptomycin. Myoblasts were transfected while the cells were in suspension 19 and studied 2 days later. PMA treatment: Myoblasts were treated with serum/antibiotic free media for 120 min and then stimulated for 20 min with PMA (100 nm in serum/antibiotic free DMEM), coincubated with/without U0126 (10 μm), BIX‐02189 (10 μm), BIM‐I (10 μm), or rapamycin (50 nm). Each series of experiments was repeated at least three times using different passages of C2C12 myoblasts.

### RNA isolation and RT‐PCR

Total RNA was prepared using TRIzol reagent according to the manufacturer's directions. RNA samples were treated with TURBO DNA‐free to remove genomic DNA contamination. Isolated RNA was quantified by spectrophotometry (λ = 260 nm). First‐strand cDNA synthesis from total RNA was performed with a mixture of oligo(dT) primer and random hexamers using SuperScript III First‐Strand Synthesis SuperMix. TaqMan real‐time RT‐PCR assays were performed using the comparative cycle threshold (CT) method with TaqMan Universal PCR Master Mix and Applied Biosystems (Foster City, CA, USA) 7300 Real‐Time PCR System instruments. Probe and primer sets for Rheb (Mm00474045_m1) and GAPDH (4352932E) were obtained from Applied Biosystems. Expression levels of each gene were determined by the 2^−ΔΔCT^ method.

### Protein extraction, immunoprecipitation, and western blotting

Samples were lysed in ice‐cold RIPA buffer [1% NP‐40, 0.5% sodium deoxycholate, 0.1% SDS, 50 mm NaCl, 20 mm Tris‐HCl (pH 7.6), 1 mm PMSF, 5 mm benzamidine, 1 mm EDTA, 5 mm *N*‐ethylmaleimide, 50 mm NaF, 25 mm glycerol 2‐phosphate, 1 mm sodium orthovanadate, and 10 μL·mL^−1^ protease inhibitor cocktail]. Lysed samples were then centrifuged at 17 860 ***g*** for 10 min at 4 °C, and the supernatants were collected for analysis. Protein concentration was determined by the Bradford method.

### Immunoprecipitation

To study the functional interactions between TSC1 and TSC2, coimmunoprecipitation assays were performed as described previously 12. CHAPS‐based buffer [0.3% CHAPS, 40 mm HEPES (pH 7.5), 120 mm NaCl, 1 mm EDTA, 10 mm sodium pyrophosphate, 10 mm glycerol 2‐phosphate, 50 mm NaF, and 10 μL·mL^−1^ protease inhibitor cocktail] was used to produce total cell lysates. One milligram of total protein was used from cell lysates and samples were immunoprecipitated with each antibody and immobilized protein A. Immunocomplexes were washed three times with CHAPS‐based buffer and then washed once with wash buffer [50 mm HEPES (pH 7.5), 40 mm NaCl, and 2 mm EDTA]. Precipitated protein samples were then subjected to SDS/PAGE.

### Western blotting

Protein extracts or immunoprecipitation samples were run on SDS/PAGE gels and transferred to polyvinylidene difluoride membrane. The membranes were blocked in Odyssey Blocking Buffer, and then incubated with dilutions of each primary antibody. IRDye 800CW Goat anti‐(mouse IgG) and IRDye 680LT Goat anti‐(Rabbit IgG) were used as secondary antibodies. Bound antibody complexes were scanned and quantified using Odyssey Infrared Imaging System (LI‐COR Biosciences).

### Subcellular fractionation

C2C12 myoblasts were washed twice with phosphate‐buffered saline and then scraped in 3 mL of ice‐cold buffer [20 mm Tricine (pH 7.8), 250 mm sucrose, 1 mm EDTA (pH 8.0), and 10 μL·mL^−1^ protease inhibitor cocktail]. The samples were homogenized using a dounce homogenizer 20 times, and then centrifuged at 1000 ***g*** for 10 min at 4 °C. The supernatant was collected and then further separated by ultracentrifugation at 300 000 ***g*** for 30 min at 4 °C. After ultracentrifugation, the pellet (membrane fraction) was directly lysed in 200 μL of 1 × SDS/PAGE sample buffer. Ten micoliters of each fractionated sample was loaded onto the gel.

### RNA interference

For knockdown of Rheb, we used predesigned siRNA reagents, Silencer Select Pre‐designed siRNA (ID: s72957) from Ambion (Foster City, CA, USA). The specific sequences of the siRNA oligonucleotide for Rheb were, sense: 5′‐CAACCAUAGAGAACACGTT‐3′ and antisense: 5′‐AACGUGUUCUCUAUGGUUG‐3′. For the transfection of siRNA oligonucleotides, we followed the procedure provided by the manufacturer. Briefly, C2C12 cells were plated at 5 × 10^4^ cells/well on six‐well culture plates, and incubated overnight. The siRNA oligonucleotides were transfected the next day at a final concentration of 50 nm using Lipofectamine 2000 transfection reagent. Three days after transfection, myoblasts samples were collected and analyzed. Knockdown efficiency of Rheb expression was confirmed by real‐time RT‐PCR (Rheb mRNA) and western blotting (Rheb protein). Silencer Select Negative Control (Ambion) was used as nonspecific transfection control.

### Statistical analysis

All results are reported as means ± SE. Student's *t*‐test was used for comparisons between groups. Multigroup comparisons were performed by two‐way analysis of variance followed by Tukey's *post hoc* test. For all comparisons, the level of statistical significance was set at *P* < 0.05.

## Results

### PMA‐induced activation of mTORC1 signaling is partially prevented by MEK inhibitors (U0126 or BIX‐02189) and blocked with mTOR inhibitor rapamycin

**Figure 1:**
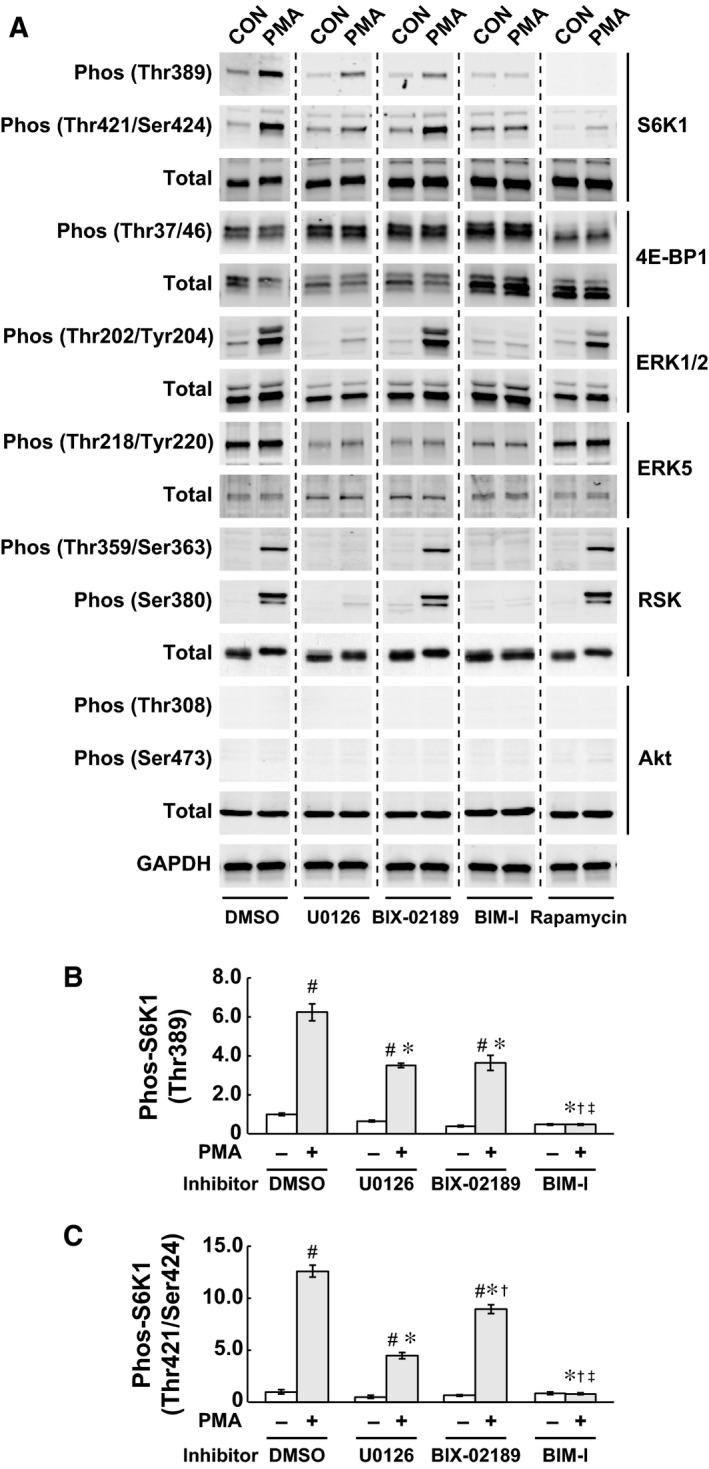
PMA‐induced activation of mTORC1 signaling is partially prevented by MEK inhibitors (U0126 or BIX‐02189) and blocked with mTOR inhibitor rapamycin. C2C12 myoblasts were treated with serum/antibiotic free DMEM for 120 min and then stimulated for 20 min with PMA (100 nm in serum/antibiotic free DMEM), coincubated with/without U0126 (10 μm), BIX‐02189 (10 μm), BIM‐I (10 μm), or rapamycin (50 nm). (A) Phosphorylation states of MEK/ERK‐dependent and mTORC1‐dependent signaling pathways were determined with using phospho‐specific antibodies (S6K1 at Thr389 and Thr421/Ser424 sites, 4E‐BP1 at Thr37/46 sites, ERK1/2 at Thr202/Tyr204 sites, ERK5 at Thr218/Tyr220 sites, RSK at Thr359/Ser363 and Ser380 sites and Akt at Thr308 and Ser473 sites, respectively). GAPDH was used as a loading control. (B) and (C) Relative phosphorylation levels of S6K1 at Thr389 (B) and Thr421/Ser424 (C) in each group were quantified. *n* = 4 in each group. All results are quantified as the fold difference compared to the unstimulated control and expressed as the mean ± SE. Significant differences: #, between unstimulated control and PMA‐treated group in each inhibitor condition (*P* < 0.05); *, from DMSO‐PMA group in each inhibitor condition (*P* < 0.05); †, from U0126‐PMA group in each inhibitor condition (*P* < 0.05); ‡, from BIX‐02189‐PMA group in each inhibitor condition (*P* < 0.05).

It has been indicated that the Ras/Raf/MEK/ERK‐dependent pathway may contribute to the regulation of muscle cell growth and hypertrophy through modulating mTORC1 signaling 14, 15. In this study, to determine the exact molecular events in the MEK/ERK‐dependent pathway leading to mTORC1 activation in skeletal muscle cells, C2C12 myoblasts were stimulated with the phorbol ester PMA, which is a well‐known agonist of protein kinase C (PKC) and its downstream effector MEK/ERK‐dependent pathway. As shown in Fig. 1, phosphorylation of S6K1 at both Thr389 and Ser421/Thr424 sites was significantly elevated (6.21 ± 0.44 fold increase in Thr389 and 12.53 ± 0.59 fold increase in Ser421/Thr424 compared to the unstimulated control, *P* < 0.05) in response to PMA treatment. In contrast, the phosphorylation status of 4E‐BP1 (Thr37/46) was not altered by PMA treatment. Inhibition of the MEK/ERK pathway by U0126, a selective inhibitor of MEK1/2, was sufficient to suppress PMA‐induced phosphorylation of S6K1 (44% decrease in Thr389 phosphorylation and 65% decrease in Ser421/Thr424 phosphorylation compared to DMSO‐PMA treatment, *P* < 0.05, Fig. 1B,C). Since U0126 potentially inhibits the MEK5/ERK5 pathway as well as ERK1/2 20, 21, a selective inhibitor of the MEK5 pathway BIX‐02189 was also used. BIX‐02189 blocked PMA‐induced ERK5 phosphorylation (46.3% decrease compared to DMSO‐PMA treatment, and almost identical to the unstimulated control) and partially prevented PMA‐induced phosphorylation of S6K1 (41% decrease in Thr389 phosphorylation and 29% decrease in Ser421/Thr424 phosphorylation compared to the DMSO‐PMA, *P* < 0.05, Fig. 1B,C). Importantly, treatment with each inhibitor did not affect fold differences in S6K1 phosphorylation compared to the unstimulated control (Thr389 phosphorylation: 6.2‐fold increase in DMSO, 5.5‐fold increase in U0126, and 9.6‐fold increase in BIX‐02189, Ser421/Thr424 phosphorylation: 12.5‐fold increase in DMSO, 8.8‐fold increase in U0126, and 12.8‐fold increase in BIX‐02189) because the basal level of S6K1 phosphorylation in unstimulated control was also diminished with inhibitor treatment. The specificity of the inhibitor treatments was confirmed as U0126 blocked PMA‐induced phosphorylation of ERK1/2 (Thr202/Tyr204) and the downstream effector RSK (Thr359/Ser363 and Ser380) but no inhibitory effects on ERK1/2 and RSK phosphorylation were observed with BIX‐02189. The specificity of the PKC agonist PMA was also confirmed as the PKC inhibitor BIM‐I completely blocked PMA‐induced phosphorylation events including S6K1, ERK1/2, ERK5, and RSK. The mTORC1‐specific inhibitor rapamycin blocked basal‐ and PMA‐induced phosphorylation of S6K1, but no inhibitory effects of rapamycin treatment on PMA‐induced phosphorylation of ERK and RSK were observed. In addition, there were no effects of PMA or inhibitor (U0126, BIX02189, BIM‐I, or rapamycin) treatment on the phosphorylation status of Akt, which is the downstream effector of PI3K signaling. These data demonstrated that PMA‐induced activation of mTORC1 signaling is mediated through parallel regulation of ERK1/2 and ERK5. In addition, it also appeared that the MEK/ERK‐dependent pathway is not an absolute requirement but, rather, contributes to mTORC1 activation upon PMA stimulation, and this signal transduction machinery is mediated independently of PI3K/Akt signaling in skeletal muscle cells.

### PMA‐induced phosphorylation of TSC2

**Figure 2:**
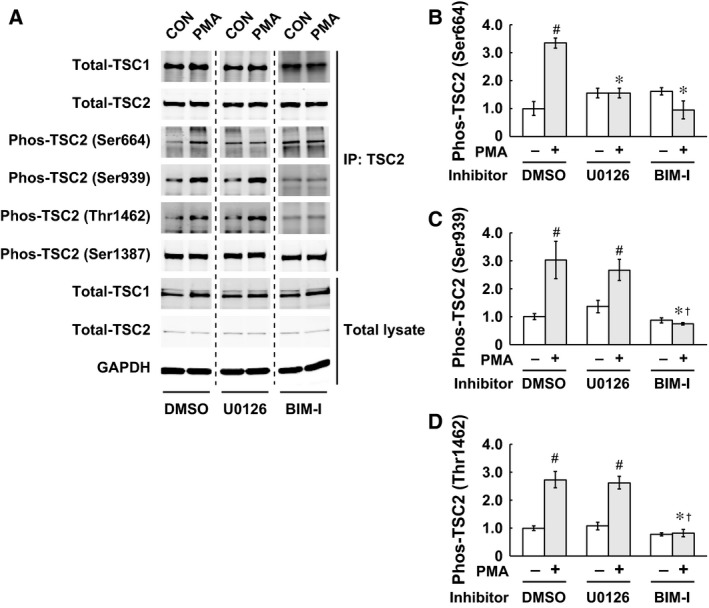
PMA‐induced phosphorylation of TSC2. C2C12 myoblasts were treated with serum/antibiotic free DMEM for 120 min and then stimulated for 20 min with PMA (100 nm in serum/antibiotic free DMEM), coincubated with/without U0126 (10 μm) or BIM‐I (10 μm). Series of the experiments were repeated at least three times using different passages of C2C12 myoblasts. Total lysates were immunoprecipitated with anti‐TSC2 antibody. TSC2 phosphorylation at each site (Ser664, Ser939, Ser1387, and Thr1462) were examined using phospho‐specific antibodies, *n* = 3–4 in each group. All results are quantified as the fold difference compared to the unstimulated control and expressed as the mean ± SE. Significant differences: #, between unstimulated control and PMA‐treated group in each inhibitor condition (*P* < 0.05); *, from DMSO‐PMA group in each inhibitor condition (*P* < 0.05); †, from U0126‐PMA group in each inhibitor condition (*P* < 0.05). TSC2 phosphorylation at Ser664, Ser939, and Thr1462 sites were increased following PMA treatment. U0126 blocked PMA‐induced phosphorylation of TSC2 at Ser664 site, but no inhibitory effect was observed at Ser939 and Thr1462 sites. TSC2 phosphorylation at Ser664, Ser939, or Thr1462 were blocked with BIM‐I. There were no changes in TSC2 phosphorylation at S1387 in response to PMA or inhibitor treatment. Coimmunoprecipitation assays showed that PMA stimulation or inhibitor treatment did not affect the physical association between TSC1 and TSC2. GAPDH was used as a loading control.

Next, we investigated protein complex formation between TSC1 and TSC2 as well as TSC2 phosphorylation status following PMA stimulation. The phosphorylation state of TSC2 was determined at the four different sites, including Ser664 (ERK‐dependent phosphorylation site), Ser939 and Thr1462 (Akt‐dependent phosphorylation sites), and Ser1387 [AMP‐activated protein kinase (AMPK)‐dependent phosphorylation site]. As shown in Fig. 2, TSC2 phosphorylation at Ser664, Ser939, and Thr1462 sites was significantly increased following PMA treatment (3.35 ± 0.19 fold increase in Ser664, 3.03 ± 0.67 fold increase in Ser939, and 2.73 ± 0.29 fold increase in Thr1462 compared to the unstimulated control, *P* < 0.05, Fig. 2A–D). The MEK1/2 inhibitor U0126 (which also potentially inhibits MEK5) blocked PMA‐induced phosphorylation of TSC2 at Ser664 site, but no inhibitory effect was observed at Ser939 and Thr1462 sites (1.95‐fold increase in Ser939 and 2.43‐fold increase in Thr1462 compared to the unstimulated control, *P* < 0.05, Fig. 2A–D). PMA‐induced phosphorylation of TSC2 at Ser664, Ser939, or Thr1462 was blocked with PKC inhibitor BIM‐I. TSC2 phosphorylation at S1387 site was unchanged in response to PMA or inhibitor treatment. Coimmunoprecipitation assays with TSC2 antibody revealed that PMA stimulation or inhibitor treatment did not affect complex formation between TSC1 and TSC2, and the amount of either TSC1 or TSC2 protein was not altered (Fig. 2A).

### mTORC1 activation through overexpression of Rheb

**Figure 3:**
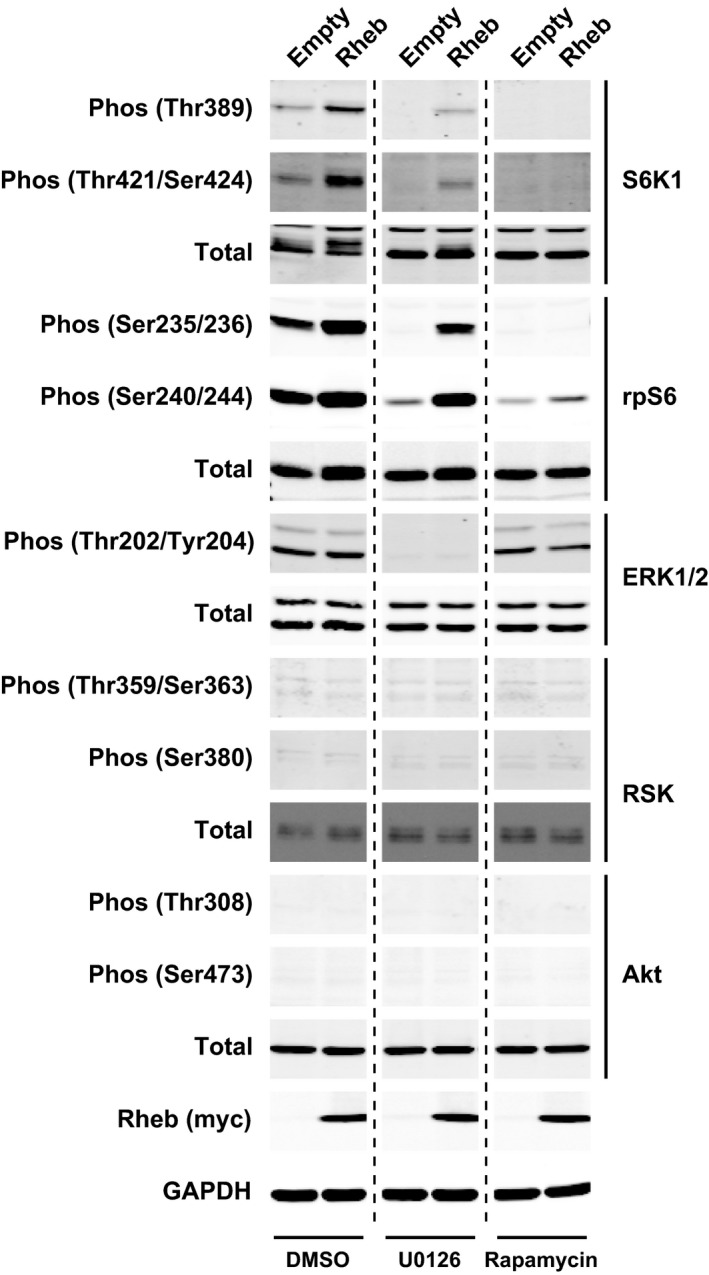
mTORC1 activation through overexpression of Rheb. C2C12 myoblasts were transfected with empty pRK5 vector or myc‐tagged Rheb. After 2 days of transfection, C2C12 myoblasts were incubated with serum/antibiotic‐free DMEM containing DMSO, U0126 (10 μm), or rapamycin (50 nm) for 120 min. Series of the experiments were repeated at least three times using different passages of C2C12 myoblasts. Phosphorylation states of MEK/ERK‐dependent and mTORC1‐dependent signaling pathways were determined using phospho‐specific antibodies (S6K1 at Thr389 and Thr421/Ser424 sites, rpS6 at Ser235/236 and Ser240/244 sites, ERK1/2 at Thr202/Tyr204 sites, RSK at Thr359/Ser363 and Ser380 sites and Akt at Thr308 and Ser473 sites). GAPDH was used as a loading control.

Although the exact regulatory mechanism remains undefined, it has been suggested that the small G‐protein Rheb is the most proximal regulator of mTORC1 activity. We examined the effects of Rheb overexpression on mTORC1 signaling. A Rheb plasmid expression vector was transfected into C2C12 myoblasts. As shown in Fig. 3, overexpression of Rheb in C2C12 cells induced a robust increase in the phosphorylation of S6K1 (Thr389 and Ser421/Thr424 sites) and its downstream effector rpS6 (Ser235/236 and Ser240/244). When myoblasts were treated with MEK inhibitor U0126, basal phosphorylation of both S6K1 and rpS6 were clearly decreased, but U0126 could not block the Rheb‐induced activation of mTORC1 signaling (as determined by the phosphorylation status of S6K1 and rpS6). In contrast, rapamycin treatment led to complete inhibition of both basal and Rheb‐induced phosphorylation of S6K1 and rpS6. No effects of Rheb overexpression were observed on the phosphorylation states of ERK1/2, RSK, and Akt. These data indicated that in skeletal muscle cells, Rheb‐induced activation of mTORC1 signaling is mediated downstream of MEK/ERK pathway.

### Rheb is required for the PMA‐induced activation of mTORC1 signaling

**Figure 4:**
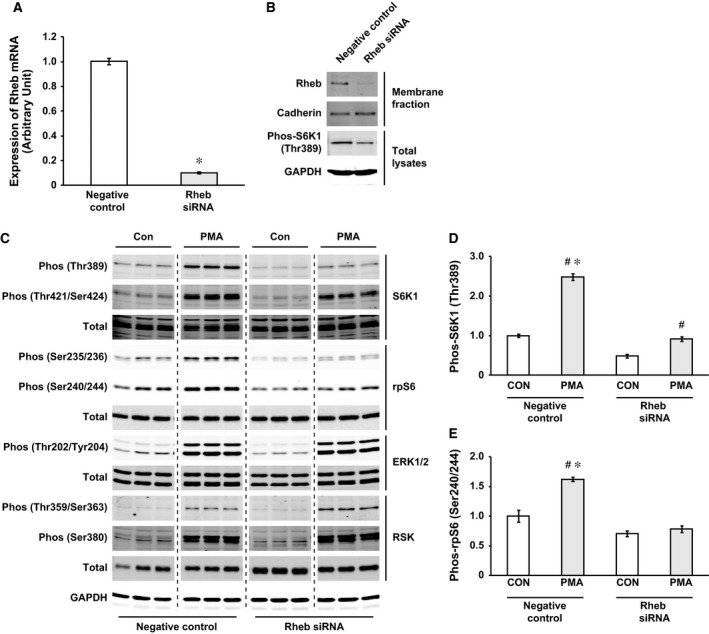
Rheb is necessary for the PMA‐induced activation of mTORC1 signaling. For knockdown of Rheb, predesigned siRNA oligonucleotides were transfected into C2C12 myoblasts. Silencer Select Negative Control was used as a nonspecific transfection control. After 3 days of siRNA transfection, myoblasts reached confluence and were used for analyses. (A) Knock down efficiency of Rheb mRNA was confirmed by real‐time RT‐PCR. GAPDH was used as an internal control for the real‐time RT‐PCR. (B) The isolated membrane fraction was used for western blotting to confirm decreased protein expression of Rheb because Rheb protein is specifically localized to the membrane fraction. Cadherin was used as an internal control for the membrane fraction samples. In the absence of Rheb, mTORC1 signaling was potently inhibited as confirmed by the decreased phosphorylation of S6K1 at Thr389 site. (C) siRNA transfected C2C12 myoblasts were treated with serum/antibiotic free DMEM for 120 min and then stimulated for 20 min with PMA (100 nm in serum/antibiotic free DMEM). (D) and (E) Relative phosphorylation levels of S6K1 at Thr389 (E) and rpS6 at Ser240/244 (E) in each group were quantified, *n* = 3 in each group. Significant differences: #, between control and PMA treated groups in each experimental condition (*P* < 0.05); *, between negative control and Rheb siRNA in each experimental condition (*P* < 0.05).

Experiments were designed to determine whether Rheb is required for the MEK/ERK‐dependent activation of mTORC1 signaling. We used siRNA oligonucleotides to knockdown endogenous Rheb levels in C2C12 myoblasts. We confirmed that knockdown efficiency of Rheb mRNA was over 90% compared to the negative control using nonsense scrambled RNA oligonucleotides (Fig. 4A). The results in Fig. 4B demonstrate that protein expression of Rheb was also diminished in cells transfected with Rheb siRNA. In the absence of Rheb, mTORC1 signaling was potently inhibited as confirmed by the decreased phosphorylation of S6K1 at Thr389 site (Fig. 4B). Next, the results demonstrated that MEK/ERK‐dependent activation of mTORC1 signaling requires the presence of Rheb in C2C12 myoblasts (Fig. 4C). As confirmed previously (Fig. 1), PMA treatment resulted in a significant activation of mTORC1 signaling, as indicated by the increased phosphorylation of S6K1 and rpS6. Conversely, in myoblasts with diminished level of Rheb, PMA treatment was not capable of the full activation of mTORC1 as determined by the blunted phosphorylation of S6K1 (67% decrease in Thr389 phosphorylation compared to the PMA negative control, *P* < 0.05, Fig. 4D) and rpS6 (complete inhibition of Ser240/244 phosphorylation in response to PMA, Fig. 4E). In contrast, we did not observe any inhibitory effects of Rheb knockdown on the PMA‐induced phosphorylation of ERK1/2 and RSK. These results demonstrated that, in C2C12 myoblasts, Rheb is required for the MEK/ERK‐dependent activation of mTORC1 signaling.

## Discussion

While the contribution of MEK/ERK‐dependent signaling to mTORC1 activation and skeletal muscle hypertrophy has been suggested 15, 16, 17, 18, the precise molecular mechanism was poorly defined. A heterodimeric protein complex TSC1/TSC2 was indicated as a central hub of signal transduction through which a variety of environmental cues were integrated to determine the functional activity of mTORC1 signaling. Growth factor‐induced activation of the PI3K/Akt pathway resulted in the TSC2 phosphorylation at multiple residues (at least two sites, Ser939 and Thr1462), which are thought to be targeted by Akt 11, 22, 23. It was also indicated that TSC2 phosphorylation at both Ser540 and Ser664 sites was directly mediated by ERK1/2 24, 25. In addition, AMPK, which is activated in response to energy stress, also phosphorylates TSC2 at Thr1271 and Ser1387 26. PI3K/Akt‐dependent, MEK/ERK‐dependent, and AMPK‐dependent pathways mediate TSC2 phosphorylation at distinct sites, indicating that these signaling pathways act independently, but possibly coordinate to control TSC2 function 27. In the present study, activation of MEK/ERK signaling, but not the PI3K/Akt pathway, resulted in a robust activation of mTORC1 signaling and increased phosphorylation of TSC2 following PMA treatment. U0126 (a selective inhibitor of MEK1/2 but which also inhibits the MEK5 pathway) treatment suppressed mTORC1 activity and prevented TSC2 phosphorylation at Ser664. This correlation strongly suggests that MEK/ERK‐dependent phosphorylation of TSC2‐Ser664 controls its GAP activity, thereby leading to mTORC1 activation. Interestingly, TSC2 phosphorylation at both Ser939 and Thr1462, two sites previously indicated as Akt‐dependent residues 11, 22, 23, was also increased in response to PMA treatment, even though no Akt activation was observed. No inhibitory effect of U0126, but complete prevention with BIM‐I treatment, was also confirmed on PMA‐induced TSC2 phosphorylation at Ser939 and Thr1462 sites. These findings suggest that a novel additional signaling axis, which is independent of both PI3K/Akt and MEK/ERK signaling but acting via PKC‐dependent signaling, regulates TSC2 phosphorylation at both Ser939 and Thr1462 sites in response to PMA stimulation. Supporting the idea that MEK/ERK signaling axis is not the sole PMA‐induced pathway of mTORC1 activation, PMA stimulation was still partially able to activate mTORC1 signaling (determined by S6K1 phosphorylation) under MEK/ERK inhibitor U0126 treatment (Fig. 1). These data suggest that cooperative phosphorylation of TSC2 at multiple sites (e.g., Ser664, Ser939, and Thr1462) may be required for control of TSC2‐GAP function and full activation of mTORC1 signaling.

The present study clearly indicates that the presence of small G‐protein Rheb, a functional target of TSC2‐GAP activity, is required for MEK/ERK‐dependent activation of mTORC1 signaling in skeletal muscle cells. We observed that, in the absence of Rheb, activity of mTORC1 signaling was significantly diminished in response to PMA treatment, although MEK/ERK signaling was fully activated. This finding is consistent with previous reports using other cell lines that Rheb is necessary for mTORC1 activation in response to environmental cues, including growth factors and nutrients 10, 13, 28. In addition, it was also reported that overexpression of Rheb in skeletal muscle was sufficient to induce mTORC1 activation and skeletal muscle hypertrophy *in vivo* 29. Collectively, the data in this study and previous reports strongly demonstrate that Rheb is an essential regulator of mTORC1 activation and enhanced protein synthesis in skeletal muscle.

Having established that MEK/ERK‐dependent regulation of mTORC1 activity is mediated through TSC2/Rheb signaling, we also need to consider that RSK, which is a downstream effector of ERK, affects mTORC1 activity through phosphorylating TSC2 at S1798, or Raptor, a component of mTORC1 30, 31. However, a recent report clearly indicated that the relative contribution of RSK‐dependent signaling to mTORC1 activation is very limited, as the RSK‐specific inhibitor did not impair the activation of mTORC1 signaling in any of the various cell types tested (except for HEK293T cells) 32. In support of this concept, it was observed in this study that siRNA knockdown of Rheb significantly inhibited mTORC1 activation, although PMA‐induced phosphorylation of RSK was completely preserved. These data suggest that RSK phosphorylation is likely dispensable for MEK/ERK‐mediated activation of mTORC1 signaling in skeletal muscle cells.

## Summary

In this study, we examined potential mechanisms involved in MEK/ERK‐dependent activation of mTORC1 signaling through TSC2 and Rheb pathways in skeletal muscle cells. The primary findings of the study were (a) treatment with PMA, a PKC agonist, and its downstream effector, the MEK/ERK pathway, resulted in robust activation of mTORC1 signaling, (b) PMA‐induced activation of mTORC1 signaling and upstream TSC2 phosphorylation (at Ser664, an ERK‐dependent site) were prevented by the MEK inhibitor U0126, (c) overexpression of small G‐protein Rheb was sufficient to activate mTORC1 signaling, and (d) PMA treatment was not capable of mTORC1 activation in the absence of Rheb in C2C12 myoblasts. These findings provide evidence that the MEK/ERK‐dependent activation of mTORC1 is mediated through TSC2 phosphorylation and its downstream effector Rheb.

## Author contributions

MM and TT conceived and designed the project and interpreted the data and wrote the paper, MM acquired and analyzed the data.
